# Assessing real-world effectiveness of therapies: what is the impact of incretin-based treatments on hospital use for patients with type 2 diabetes?

**DOI:** 10.1186/s13561-022-00397-5

**Published:** 2022-10-22

**Authors:** Clémence Bussiere, Pauline Chauvin, Jean-Michel Josselin, Christine Sevilla-Dedieu

**Affiliations:** 1 ERUDITE (CNRS-EA437), University of Paris Est Créteil, Paris, France; 2 MGEN Foundation for Public Health, Paris, France; 3 LIRAES (URP4470), Université Paris Cité, F-75006 Paris, France; 4 CREM (CNRS-UMR6211), University of Rennes 1, Rennes, France; 5 Centre des Saints-Pères, 45 rue des Saints-Pères, 75006 Paris, France

**Keywords:** Diabetes, Drug assessment, Hospital use, Quasi-experiment, Observational data, I10, D61

## Abstract

**Background:**

Managing type 2 diabetes represents a major public health concern due to its important and increasing prevalence. Our study investigates the impact of taking incretin-based medication on the risk of being hospitalized and the length of hospital stay for individuals with type 2 diabetes.

**Method:**

We use claim panel data from 2011 to 2015 and provide difference-in-differences (DID) estimations combined with matching techniques to better ensure the treatment and control groups’ comparability. Our propensity score selects individuals according to their probability of taking an incretin-based treatment in 2013 (N = 2,116). The treatment group includes individuals benefiting from incretin-based treatments from 2013 to 2015 and is compared to individuals not benefiting from such a treatment but having a similar probability of taking it.

**Results:**

After controlling for health-related and socio-economic variables, we show that benefiting from an incretin-based treatment does not significantly impact the probability of being hospitalized but does significantly decrease the annual number of days spent in the hospital by a factor rate of 0.621 compared with the length of hospital stays for patients not benefiting from such a treatment.

**Conclusion:**

These findings highlight the potential implications for our health care system in case of widespread use of these drugs among patients with severe diabetes.

**Supplementary Information:**

The online version contains supplementary material available at 10.1186/s13561-022-00397-5.

## Background

The prevalence of Type 2 Diabetes (T2D) is high and steadily increasing worldwide. In France, nearly 5% of the adult population is enrolled in an anti-diabetic treatment [1]. Anti-diabetic treatments aim at improving glycemic control to avoid complications related to T2D. The most common drug adverse event related to glucose-lowering therapies is hypoglycemia. It increases the risk of falls, major adverse cardiovascular events, dementia and mortality [2, 3]. The therapeutic strategy for T2D has been limited for decades to insulin, metformin, and sulfonylureas (traditional treatments). Newer glucose-lowering agents, namely incretin-based treatments (IBT), have been marketed since 2006–2007 in France [4]. They include inhibitors of dipeptidyl peptidase-4 (incretin enhancers, DPP4i) and glucagon-like peptide-1 analogs (incretin mimetics, GLP1a). Both drugs demonstrate improvements in glucose control with minimal risk of hypoglycemia [5–7]. However, these medications are more expensive than older drugs [4, 8]: in 2012 IBT represented 15% of prescription drugs for managing T2D and amounted to 50% of these expenditures [9]. In addition, even if improvements in patients’ health thanks to IBT have been demonstrated with respect to a limitation of side effects and short-term health indicators in randomized trials, uncertainty remains when considering their effectiveness and efficiency in a real-life setting [10, 11].

More generally, pharmaceutical innovation is a major cause of rising drug-related health expenditures [12]. However, these increases may be at least partially offset by decreases of other health care consumptions [13]. Real-world data are an important information source for health care regulators to assess the actual impact of medical treatments. Indeed, these data enable to include a wider range of patients and more patients with comorbidities and advanced age (who are often excluded from randomized clinical trials) and consider longer observation periods. However, analyzing these data is challenging since they are not based on randomly assigned groups.

Our study aims to complement previous investigations of the relationship between IBT and hospital use which yielded mixed results [14, 15]. Knowing whether IBT can change hospital uses of T2D patients is an important issue since hospitalizations are the first cost driver of this population (mostly due to complications [16, 17]).

Since IBT are more expensive than traditional anti-diabetic treatments, investigating whether the additional cost associated with prescribing them is offset by decreases in hospital uses would contribute to the assessment of the efficiency of these drugs. In addition, evaluating IBTs’ impact would contribute to the debate regarding their use with respect to traditional treatments for glycemic control. Indeed, no consensus currently exists regarding this topic: the French HTA body (HAS) recommends prescribing them as a third-line treatment, while ADA-EASD (American Diabetes Association and European Association for the Study of Diabetes) advocated for using them as an add-on therapy to first-line metformin to minimize hypoglycemia [18, 19]. Meanwhile, IBT are significantly more frequently prescribed in France compared to most European countries [9] or Australia [20].

The paper is organized as follows. The next section describes our data and details our identification strategy, which is preceded by a brief reminder of recommendations for treating T2D patients and finishes with statistical analysis. The results are then presented and discussed before we conclude.

## Methods

### Data

Our study is based on claims data from MGEN (Mutuelle Générale de l’Éducation Nationale). This leading French mutual provides statutory health insurance (NHI) for employees in public education, culture, research and sport (both while working and after retirement) and also offers voluntary complementary health insurance coverage.

Our sample is built from the ADAM (Analyse sur les Diabétiques Assurés de la Mutuelle) cohort which was already used in previous studies (e.g., [21, 22]). This cohort includes 80,989 enrollees who were at least 18 years old and had received at least one anti-diabetic medication (oral agents or insulin) in 2012. The prevalence of diabetes among the MGEN population is approximately 3%, which is slightly lower than that observed in France, which was 5% in 2015 [23]. Comprehensive health reimbursement data and sociodemographic characteristics (such as age, gender, place of residence, contribution base – similar to income, and eligibility for 100% coverage) are available for ADAM cohort participants only when they are covered by both NHI and MGEN complementary health insurance.

We selected from ADAM cohort individuals for whom complete information over the period 2011–2015 is available and who have at least one claim for an anti-diabetic treatment other than insulin each year. This latter criterion ensures that most Type 1 diabetic individuals whose treatment is exclusively based on insulin are excluded, as well as incidental cases of diabetes over the period 2012–2015. This panel dataset gathers for the selected individuals their individual characteristics (i.e., health-related, sociodemographic and environmental variables) and their quantity of individual health care consumptions on a yearly basis from 2011 to 2015.

### Identification strategy

Using real-world data to measure IBTs’ impact is challenging due to the non-random assignment of treatments, which requires using quasi-experimental designs. The therapy scheme for T2D follows a sequence, which starts with a monotherapy (one molecule), then, as the disease worsens, a dual therapy (two molecules), followed by a triple therapy (three molecules) and ultimately, an insulin therapy. Managing T2D can be complex and recommendations may differ according to sources [10]. The HAS recommends prescriptions that combine IBT in a dual therapy or triple therapy only in cases of contraindications to or intolerance of traditional treatments (e.g., metformine, sulphonylurea) or when these latter therapies do not meet glycemic targets [19]. Meanwhile, the ADA-EASD and the Francophone Diabetes Society (SFD) promote prescribing IBTs for patients with specific conditions such as chronic kidney or cardiovascular diseases [18, 24].

If prescribers strictly comply with the HAS recommendation [25], a naive comparison of hospital use and length of stay between patients using IBT and those using traditional treatments is likely to be biased, since the first group is likely to include patients with more advanced and complicated diabetes on average. Meanwhile, in clinical practice, IBT are given to a much broader population than the one targeted in the HAS recommendation. For instance, from our ADAM sample, approximately 5% of patients use IBT alone. Computing IBTs’ impact only using the recommended population would not enable measuring these treatments’ actual impact on the T2D population.

#### Quasi-experimental design to tackle selection biases

Several alternative methods exist for estimating the impact of a new treatment. Among them, statistical methods for survival analysis (e.g. Cox model [26]) play a central role in the assessment of treatment effects, mostly in randomized clinical trials.

Real-life data requires using a quasi-experimental design when measuring treatment impacts in order to control for selection biases due to non-random treatment assignation (so that differences between treated and non-treated individuals can be attributed to the treatment itself). We use the difference-in-difference (DID) method [27–30] to control for observed and unobserved differences between the treatment and control groups that are constant over time. DID method requires a panel dataset with two periods (before and after introducing treatment) and two stable groups (treatment and control).

Our models estimate differences in changes in average hospital use and length of stays over time between patients taking traditional drugs and patients starting IBT during the study period. As our study period spans five years (from 2011 to 2015), we needed at least two years before the introduction of the treatment to compare trends in hospital use between the two groups. We extracted an initial sample (N = 26,244) for which the type of antidiabetic treatments only changed in the treatment group and only once (during 2013 – the reference year). The two groups of individuals are the following: (1) the treatment group, composed of individuals under traditional treatment from 2011 to 2012, initiating an IBT in 2013 and keeping this type of treatment in both 2014 and 2015 and (2) the control group, which gathered individuals who never had an IBT and who benefited from traditional treatment from 2011 to 2015.

The DID method does not require similar hospital use between groups before introducing the treatment but similar trends [30]. To be able to compare groups with similar trends, we combine the DID estimations with propensity score matching (PSM) [28]. The PSM approach aims to balance the treatment and control groups according to pre-treatment outcomes and covariates to control for time-invariant residual biases [29]. The process generates a subsample by matching individuals according to their probability of taking an IBT in 2013. Our main analysis is based on the following matching (model 1 – M1): individuals in the treatment group are matched one to one (1:1) to individuals in the control group from their estimated propensity score using the nearest neighbor matching estimator [31] without replacement and with a caliper, which imposes a maximum tolerated difference between matched subjects of 0.25 standard deviation of the propensity score [32]. Because starting an IBT theoretically depends on a predefined therapeutic scheme, the following matching variables are included in the logistic estimation of the propensity score: age, type of therapy (dual, triple or insulin therapy), endocrinologist visit, polypharmacy indicator, and previous hospital admissions (see next section for details about these variables). Preliminary bivariate analysis was conducted to ensure that these variables were relevant indicators of the probability of taking an IBT. The quality of the resulting matched samples is assessed through tests for equality of means in the treated and control groups, both before and after matching (Appendix B). The standardized bias (difference in means of each covariate divided by the standard deviation) is used as a balance indicator [33], targeting a value below 5% after matching [34].

Because the matching process depends on matching algorithms, we check the robustness of our results with alternative processes. We first define an alternative propensity score including different covariates (model 2 – M2). We also conduct a matching that pairs individuals with the same value of observed characteristics for binary or categorical variables (exact matching) and finds the “nearest” neighbor for continuous variables (weighted function) (model 3 – M3). Finally, we test a k-nearest neighbor matching method. This method is expected to decrease sampling variance thanks to a larger matched sample size [35]. However, it may be less effective in eliminating selection biases between the compared groups. We display a 1:5 matching with caliper and without replacement where one treated participant is matched to 5 controls (model 4 – M4).

### Statistical analysis and description of variables

The DID estimations are conducted in the matched sample. The regression specification is as follows:

$$\begin{gathered} {E_{it}}=\alpha +\beta {X_{it}}+{\gamma _0}Pos{t_{it}}+{\gamma _1}Treatmen{t_{it}} \\ +{\gamma _2}DI{D_{it}}+{c_i}+{\varepsilon _{it}} \\ \end{gathered}$$

Where $i$ and $t$ index individuals and time periods, respectively; $\alpha$, $\beta$,${\gamma }_{0}$, ${\gamma }_{1}$, and ${\gamma }_{2}$ are parameters to be estimated; ${c}_{i}$ denotes unobservable time-invariant individual heterogeneity and ${{\varepsilon }}_{\text{i}\text{t}}$ is the random error term. ${\text{E}}_{\text{i}\text{t}}$ refers to variables measuring hospital utilization. Hospital admissions here refer to all-cause hospitalizations. The two considered outcomes are: (1) a dichotomous variable coded as 1 if patient $i$ was admitted at least once to a hospital in year $t$ and 0 otherwise and (2) a discrete variable indicating the number of inpatient days for patient $i$ in year $t$. $Post$ is a dummy variable coded as 1 if observations are collected during the post-period (from 2013) and 0 otherwise, $Treatment$ indicates the treatment group and $DID$ is the interaction term ($Post\times Treatment$). The coefficient of the DID term captures the causal effect of IBT on hospitalization. The covariates from vector $X$ include health-related, sociodemographic and the environmental variables.

The explanatory variables are selected from the rationale of the health capital model [36] and from preliminary binary statistical analyses. Indeed, many stylized facts in the literature provide support for using the health capital model in the case of diabetic patients [22, 37–39]. We describe and justify the selected variables in the rest of the paragraph. First, hospital consumption depends on patients’ health states. We expect that the healthier patients are, the less likely they are to be hospitalized. We use indicators of health services as a proxy for health status because no direct measure occurs in our claims data. Dummies indicating hospital use in the previous year and the type of therapy (mono-, dual-, triple-, insulin-) provide proxies for diabetes severity; a continuous polypharmacy variable accounts for accumulated drugs prescribed at least three times in the year [40] apart from anti-diabetic treatments, which provides proxies for a patient’s comorbidities. Second, an aggregate score of recommended medical follow-up and a variable indicating at least one visit to an endocrinologist during the year are used as proxies for the patient’s observance in managing his or her diabetes. The medical follow-up score includes eight items including regular controls for blood pressure and lipids, regular screenings for eye damage, and influenza vaccinations [25]. The score ranges from 0 to 1 according to the patient’s adherence to the eight items. Individuals with poor adherence to follow-up recommendations experience a higher depreciation in their health capital, resulting in a higher risk of complications and consequently inpatient care. Third, for a given health state, we can expect different levels of hospital use depending on patients’ sociodemographic backgrounds. The sociodemographic variables are age, marital status, employment status, 100% coverage status granted by the social health care program for chronic illnesses and income. Finally, we consider environmental variables: at the local level, whether the location is in a rural area or not, and at the county level, the share of diabetes-related deaths (as a proxy for the local prevalence of diabetes, which reflects the local characteristics of health care needs) and the density of hospital beds (as a proxy of market competition and healthcare accessibility). The latter variable describes market competition and health care accessibility in a given geographical area that can explain differences in hospital uses across individuals.

We conduct our estimations as follows. First, a linear probability model (LPM) predicts the probability of being hospitalized at least once during the year. We choose a LPM because the interaction effect is mismeasured in binary models (logit or probit): the process of statistical testing about partial effects, and interaction terms in particular, may produce uninformative and sometimes contradictory and misleading results in these models [41–44]. We use the Stata command *robust* to estimate proper standard errors. Second, a hurdle model analyzes the number of inpatient days. The hurdle specification is a conditional Poisson model [44]. It handles two-part distributions and empirical frequencies of zero [45]: one distribution addresses the zeros (i.e., hospital use likelihood), while another distribution addresses the positive nonzero counts (i.e., length of stay conditional on at least one hospital admission). Results with a zero-inflated Poisson (ZIP) regression, another mixture model for two-part distributions, are provided (model 5 – M5). Analyses are conducted using Stata 14.0.

## Results

### Descriptive statistics

Descriptive analyses before and after the matching are presented for the year 2013 in Table 1.

**Table 1:** Descriptive statistics for the year 2013

		Unmatched sample	Matched sample		
			All	Treated group	Control group
***Variables***		N = 26,244	N = 2,116	N = 1,058	N = 1,058
**Health-related**					
Incretin-based treatment		6.09	50.00	100.00	0.00
Hospital use in the previous year		16.36	20.35	21.37	19.38
Number of days in hospital^†^		9.69 ± 17.34	10.11 ± 16.06	10.53 ± 14.62	9.66 ± 17.48
At least one GP visit		97.06	96.55	96.30	96.80
Number of GP visits^†^		6.90 ± 5.58	7.72 ± 4.69	7.49 ± 5.02	6.95 ± 4.32
At least one endocrinologist visit		6.54	14.10	13.88	14.31
Number of endocrinologist visits^†^		2.08 ± 1.29	2.19 ± 1.20	2.22 ± 1.08	2.17 ± 1.30
Type of therapy for diabetes					
	Monotherapy	59.11	1.95	1.94	1.96
	Dual therapy	25.79	47.19	48.01	46.40
	Triple therapy	4.55	34.00	35.06	32.98
	Insulin therapy	10.55	16.86	14.99	18.67
Polypharmacy		7.00 ± 4.10	7.24 ± 4.09	7.21 ± 4.23	7.28 ± 3.95
Diabetes follow-up (score)		0.55 ± 0.22	0.58 ± 0.21	0.59 ± 0.21	0.57 ± 0.22
**Sociodemographic**					
Age		71.72 ± 9.35	69.94 ± 9.28	69.84 ± 9.19	70.85 ± 9.37
Female		48.12	44.33	48.01	40.80
Living with partner		69.59	69.49	68.55	70.40
Working		13.02	14.64	14.06	15.20
100% coverage status		85.08	91.30	89.18	93.33
Income		2227.80 ± 1459.41	2254.11 ± 1936.50	2224.07 ± 1016.14	2282.98 ± 2522.46
**Patient’s environment**					
Location in a rural area (local level)		5.69	5.39	5.27	5.51
Share of diabetes-related deaths (county level)		2.13 ± 0.68	2.18 ± 0.71	2.16 ± 0.65	2.20 ± 0.77
Density of hospital beds		642.69 ± 160.71	642.63 ± 154.13	645.34 ± 145.95	640.23 ± 162.81

Note. (%) or (mean ± sd)

^†^Among individuals who were experienced the event at leat once (i.e., hospitalization or GP visit or endocrinologist visit)

The matched sample contains 2,116 individuals. The mean age in our matched sample was 69.94 years, 44.33% were women, 14.64% were active, and 91.30% benefited from 100% coverage. Most of them had at least one GP visit (96.97%), 14.10% visited an endocrinologist and took on average seven prescribed drugs. 20.35% were hospitalized at least once in the previous year with an average length of stay of 10.11 days. A total of 69.49% lived with a partner, and 5.39% lived in a rural area.

Compared with before the matching, our matched sample gathers younger individuals, with more severe diabetes (less monotherapy and more hospital stays). They have higher score of recommended medical follow-up, visit more often endocrinologist at least once a year. They also benefit more frequently from 100% coverage and consume more prescribed diabetes-unrelated drugs.

More results about the matching process are given in Appendix 1. After matching, covariates used for the matching have similar distributions between control and treatment groups. No significant differences in trends in hospital use and length of stay between 2011 and 2013 are observed after matching regarding hospital admission ($$p=0.2113$$) and length of stay when admitted ($$p=0.5687$$). Before matching, differences were significant ($$p=0.0771$$and $$p=0.0516)$$.

### Estimates from the main model

The results from our main regression analysis (M1) are presented with linear coefficients for hospitalization likelihood and incidence-rate ratios (IRRs) for the length of stay, with 95% confidence intervals (Table 2).

**Table 2:** Regressions among the matched sample (N = 2,116)—Model M1

		Probability of being hospitalized					Length of stay				
		Linear probability model					Zero truncated Poisson^†^				
***Variables***		***Coeff***		***95%CI***			***IRR***		***95%CI***		
*DID*		-0,01		-0,04	-	0,03	0,62	***	0,57	-	0,68
Post period		0,04		0,00	-	0,07	1,24	***	1,24	-	1,35
Treatment (Incretin-based treatment)		1^‡^					1^‡^				
hospital use in the previous year		0,13	***	0,11	-	0,16	1,03		0,98	-	1,07
At least one endocrinologist visit		0,05	**	0,00	-	0,10	1,03		0,93	-	1,15
Type of therapy for diabetes											
	Monotherapy	Ref					Ref				
	Dual therapy	0,01		-0,02	-	0,04	1,35	***	1,25	-	1,46
	Triple therapy	0,03		-0,01	-	0,07	1,61	***	1,46	-	1,77
	Insulin therapy	0,19	***	0,13	-	0,26	1,98	***	1,80	-	2,18
Diabetes follow-up (score)		-0,05	**	-0,09	-	-0,02	0,83	**	0,74	-	0,93
Polypharmacy		0,04	***	0,04	-	0,05	1,03	***	1,02	-	1,03
Age		0,00	**	0,00	-	0,00	1,04	**	1,01	-	1,07
Living with partner		0,00		-0,02	-	0,01	1,05		0,55	-	2,03
Working		-0,03		-0,03	-	0,05	0,77	*	0,58	-	1,01
100% coverage status		0,04	**	-0,11	-	0,03	1,66	***	1,37	-	2,00
Ln(income)		-0,04		-0,01	-	0,02	1,42	**	1,08	-	1,86
Location in a rural area		-0,01		-0,24	-	0,23	0,50	**	0,27	-	0,94
Share of diabetes-related deaths		-0,02		-0,05	-	0,02	1,55	**	1,33	-	1,81
Density of hospital beds		0,00		0,00	-	0,00	1,01	***	1,01	-	1,01

Notes. Coeff = coefficient of the linear probability model; IRR = incidence rate ratios; 95%CI = 95% confidence intervals; * p < 0.1, ** p < 0.05, *** p < 0.0001

^†^Second part of the Hurdle model, i.e. the number of days hospitalized conditionally to at least one hospital admission

^‡^Effect of treatment omitted because of no within group variation

Overall, our results indicate that adopting an IBT does not significantly affect the likelihood of being hospitalized but reduces the length of stay. After hospital admission, individuals having an IBT stay on average 0.621 times less long at hospital than those having a standard treatment.

Overall, covariates have the expected signs. The following ones are significantly and positively associated with a higher level of hospital use for the two outcomes: increasing age, taking insulin compared to monotherapy, being subject to polypharmacy, and having a 100% coverage status. We also find the expected negative association with the annual follow-up recommendation score. However, being hospitalized the year before is significantly associated with the probability of hospitalization only. Dual, triple and insulin therapies compared with monotherapy are significantly associated with an increasing number of inpatient days. The number of inpatient days is lower among active individuals and is positively associated with income increases. Finally, the length of stay is negatively associated with being located in a rural area and positively with the county share of diabetes deaths and the county density of hospital beds.

### Robustness checks

Table 3 summarizes the results obtained from four alternative models (M2 to M5).

**Table 3:** Summary of results according different model specifications and estimation techniques—(Models M1 to M5)

				Probability of being hospitalized						Length of hospital stay				
				Linear probability model						Zero truncated Poisson^†^				
				***Coeff (DID)***		***95%CI***				***IRR (DID)***		***95%CI***		
**Changes in the matching variables**:			**M2**	-0,02		-0,05	-	0,02		0,72	***	0,66	-	0,79
	Socioeconomics: age, gender, partnership, employment situation, coverage eligibilty													
**Changes in the matching method**:														
	Exact matching		**M3**	0,01		0,42	-	1,29		0,73	***	0,65	-	0,93
	k-nearest (k = 5)		**M4**	-0,01		-0,04	-	0,01		0,74	***	0,69	-	0,79
**Changes in the Two Part version model**:			**M5**							0,82	***	0,74	-	0,91
	Estimation of a ZIP model (results for the second part)													

Notes. Coeff = coefficient of the linear probability model; IRR = incidence rate ratios; 95%CI = 95% confidence intervals; The models are controlled for the full same set of covariates (see Table 3);*** p < 0.0001

†Second part of the Hurdle model, i.e. the number of days hospitalized conditionally to at least one hospital admission

First, alternative matching processes (M2 to M4) are tested. Whatever the matching process, we reach the same conclusions: there is no significant effect of IBT on the probability of being hospitalized, but a significant decrease in the length of stay occurs if admitted. Second, the ZIP regression (M5) confirms our initial results.

## Discussion

This study case illustrates the usefulness of using real-world data to demonstrate the effects of change in drug treatments on other health care uses, especially when prescribers do not systematically follow clinical recommendations. Our results show that IBT has no significant impact on the probability of being hospitalized but significantly shortens hospital stays.

To the best of our knowledge, the present study is a unique application of the DID methodology combined with matching using panel administrative data to evaluate the impact of IBT on the utilization of health services in diabetes treatment. Previous studies [15, 46] have also employed DID models to appreciate the impact of another innovative anti-diabetic treatment. The DID approach allowed to control for time invariant unobserved heterogeneity. This is especially important for our purpose and our population of interest since these unobserved characteristics may impact individuals’ health (such as behaviors related to lifestyle, time preferences, etc.). The matching helped to improve the comparability between control and treatment groups to further investigate the causal impact of these IBTs. Several sensitivity analyses confirmed our conclusion’s robustness regarding the matching procedure and the regression model’s specification. Since IBT is proven to limit hypoglycemia risk [47], we argue that the lower length of stays among patients benefiting from IBT may be explained by less severe episodes when hospitalized compared to similar patients undergoing standard treatments.

Concerning the impacts of other covariates, we did not find any untenable results: increasing age and other proxies of worse health status are associated with higher hospital use, whereas better adherence to follow up guidelines decreases hospital use. Increasing length of stay for patients in dual, triple or insulin therapies compared to monotherapy may be interpreted in terms of the diabetes’s duration and severity. The positive association between the length of stay and both the 100% coverage status and income can be interpreted as a greater ability to pay for hospital charges. Finally, the positive correlation between the patient’s environment and the length of stay is presumably attributed to health care organization.

Our results complement and is rather in line with the literature that addresses IBTs’ impact on alternative health care uses. Detournay et al. demonstrated that hospitalization due to severe hypoglycemia and all-cause emergency visits were significantly less frequent among patients treated with DPP4i versus sulfonylurea or glinides in France [48]. Alternatively, using a DID methodology, an America [46] and a Taiwanese [15] study found contradictory results about the impact of introducing an IBT on total health care expenditures. Balkrishnan et al. showed that American patients newly treated with IBT had lower total annual health care costs compared to patients newly treated with traditional anti-diabetic drugs [46]. Conversely, Liu et al. observed that adopting IBT increased pharmaceutical expenditures but did not change the hospital admission rate, the length of hospital stays in Taiwan nor other outpatient and inpatient expenditures [15]. These results must nevertheless be compared to ours with caution since the class of IBT they considered (thiazolidinedione) was not included in our study as this therapeutic class could not be prescribed in France during our period of interest. In line with Liu et al. [15], we did not measure a significant change in hospitalization probability. Simultaneously, as found in Detournay et al. [48] and Balkrishnan et al. [46], our study shows a decrease in the length of stays when hospitalized.

Our study has nevertheless some limitations. First, the scope of our conclusion does not refer to the average T2D population but to a subpopulation in which individuals are likely to undergo an IBT. These patients suffer from more severe diabetes than the average T2D population. Second, our database does not encompass individuals’ health status such as glycemic control. Rather, we used proxies of health as commonly performed when using administrative data. Third, we could not identify diabetes-related hospitalizations. However, diabetes complications were demonstrated to represent a large proportion of all hospitalizations of diabetic patients [49, 50]. Fourth, using claims data only gives information about drug delivery. We nevertheless used an aggregate score of recommended medical follow-up and a variable indicating at least one visit to an endocrinologist during the year to partially capture information about patients’ observance in the management of their disease. Finally, the ADAM cohort was built from the MGEN population who is on average more educated and wealthier than the French general population. These differences can explain a slightly lower percentage of diabetes compared with the general population (3% versus 5%). This population is also expected to take better care of itself, to be more observant on hygiene and dietary recommendations, which may lead to underestimate the reduction of hospital use due to IBTs. However DID estimations controlled for unobserved characteristics that do not vary over the period of observation.

## Conclusion

Our study shows that IBT for T2D treatment do not lower hospitalization risk but reduce the duration of hospital stay when individuals are hospitalized. These findings highlight the potential implications for our health care system in case of extended use of these drugs among patients with severe diabetes. The decrease in the length of stay when hospitalized is expected to reduce diabetic patients’ hospital costs, which is the largest health expenditure for this population. Beyond budgetary considerations, a shorter length of stay can also be interpreted as patients having less acute hypoglycemic episodes and thus better quality of life.

These results contribute to better inform health policy stakeholders on the consequences of introducing incretin-based treatments in the debate of updating recommendations regarding drug strategy for glycemic control. Overall, the results raise awareness concerning the usefulness of health-impact studies based on real-world data to assess newer therapies.

## Electronic supplementary material

Below is the link to the electronic supplementary material.

Supplementary Material 1

## Data Availability

Data analyzed during the current study are stored at the MGEN Foundation for Public Health, France. They are not publicly available.
