# Supplementary material for: Assessing real-world effectiveness of therapies: what is the impact of incretin-based treatments on hospital use for patients with type 2 diabetes?
Source: Health Econ Rev. 2022 Oct 22;12:53. doi: 10.1186/s13561-022-00397-5 (PMC9587565; doi:10.1186/s13561-022-00397-5)
Supplement: Supplementary file 1 — Supplementary Material 1 [file 13561_2022_397_MOESM1_ESM.docx]

| Appendix 1. Balancing before and after matching |  |  |  |  |  |  |
| --- | --- | --- | --- | --- | --- | --- |
|  | Unmatched (N=26,244) | % bias | % reduction \|bias \| | t-test^†^ | |  |
| *Variables* | Matched (N= 2,116) |  |  | t | p>\| t \| |  |
|  |  |  |  |  |  |  |
| Age | Unmatched | -26,8 |  | -7,79 | 0,00 |  |
|  | Matched | 3,5 | 86,7 | 1,30 | 0,41 |  |
|  |  |  |  |  |  |  |
| Type of therapy for diabetes: dual therapy | Unmatched | 36,7 |  | 13,10 | 0,00 |  |
|  | Matched | 1,6 | 95,5 | 0,39 | 0,73 |  |
|  |  |  |  |  |  |  |
| Type of therapy for diabetes: triple therapy | Unmatched | 119,9 |  | 91,75 | 0,00 |  |
|  | Matched | -0,3 | 99,8 | 0,00 | 0,96 |  |
|  |  |  |  |  |  |  |
| Type of therapy for diabetes: insulin therapy | Unmatched | 7,3 |  | 4,67 | 0,01 |  |
|  | Matched | -2,1 | 71,5 | -0,51 | 0,68 |  |
|  |  |  |  |  |  |  |
| Polypharmacy | Unmatched | 36,7 |  | 17,28 | 0,00 |  |
|  | Matched | -1,8 | 95,1 | -0,44 | 0,71 |  |
|  |  |  |  |  |  |  |
| At least one endocrinologist visit | Unmatched | 5,7 |  | 2,89 | 0,04 |  |
|  | Matched | 1,7 | 71 | 0,63 | 0,70 |  |
|  |  |  |  |  |  |  |
| At least one previous hospital admission (year 2012) | Unmatched | 5,9 |  | 2,36 | 0,02 |  |
|  | Matched | 1,0 | 83,0 | 0,42 | 0,81 |  |
|  |  | MeanBias | MedianBias |  |  |  |
|  |  |  |  |  |  |  |
| Sample | Unmatched | 33,8 | 26,8 |  |  |  |
|  | Matched | 1,7 | 1,7 |  |  |  |
| ^†^Tests for equality of means in the treated and control groups, both before and after matching to assess the quality of the matching. | | | | | |  |
